# Prognostic factors of pediatric ependymomas at a National Cancer Reference Center in Peru

**DOI:** 10.3389/fonc.2023.1331790

**Published:** 2024-01-17

**Authors:** Eduardo Perez-Roca, Tatiana Negreiros, Sandro Casavilca-Zambrano, Luis Ojeda-Medina, Rosdali Díaz-Coronado

**Affiliations:** ^1^ School of Medicine, Universidad Peruana Cayetano Heredia, Lima, Peru; ^2^ Radiotherapy Department, Instituto Nacional de Enfermedades Neoplásicas, Lima, Peru; ^3^ Pathology Department, Instituto Nacional de Enfermedades Neoplásicas, Lima, Peru; ^4^ Neurosurgery Department, Instituto Nacional de Enfermedades Neoplásicas, Lima, Peru; ^5^ Pediatric Oncology Department, Instituto Nacional de Enfermedades Neoplásicas, Lima, Peru

**Keywords:** pediatric, ependymoma, treatment, prognosis, outcomes, Peru

## Abstract

**Background:**

Ependymomas are central nervous system tumors that significantly impact the quality of life and carry a high mortality rate. Both the disease itself and its treatment cause significant morbidity. At a national level in Peru, there are no reports on clinical characteristics of the disease.

**Methods:**

This retrospective study captured patient aged less than 19 years with a diagnosis of ependymoma from 2012 to 2022 at a tertiary center in Lima.

**Results:**

85 patients were included with a median follow-up time was 51.6 months. The 5-year overall survival and progression-free survival were 55.89% (95% CI: 44.28 – 65.99) and 37.71% (95% CI: 26,21-49,16) respectively. The main prognostic factors identified were completed treatment (p=0.019), adjuvant chemotherapy (p=0.048), presence of metastasis (p=0.012), and disease recurrence (p=0.02).

**Conclusions:**

The survival of patients with ependymoma is below that reported in high-income countries. Incomplete treatment and treatment abandonment are factors that negatively impact the prognosis. Further studies are needed to identify barriers in the referral and treatment process for patients with ependymoma.

## Introduction

Brain tumors constitute the second most prevalent form of pediatric cancer, with ependymomas comprising 4.6% of them (1). According to Lima’s Cancer Registry, a population-based registry that best represents the incidence of different cancers in Peru, between 2013 and 2015, 26 cases of pediatric ependymomas in patients younger than 15 years of age were documented, with a frequency of 8.6 cases per year in the aforementioned time period (2). For patients diagnosed with ependymoma, the disease and its treatment cause significant morbidity, affecting short-term and long-term development (3–7).

Neurosurgical resection and radiation therapy are considered the cornerstones of ependymoma treatment, achieving the highest overall survival (OS) and progression-free survival (PFS) rates (8–10). The role of chemotherapy is still under investigation, as consistent benefits have not been reported (11). Therapeutic alternatives such as adjuvant chemotherapy and radiation therapy or the omission of adjuvant therapy may be valid options for certain patient subgroups, depending on clinical and molecular features that have yet to be characterized (12).

Historically, the classification of ependymomas was based solely on their histological characteristics. Specifically, the anaplastic subtype (grade 3) has been associated with a poorer prognosis, although these findings have not been consistent across different studies. Furthermore, high interobserver variability and low reproducibility limit its application (13, 14). In the last decade, molecular characterization of these tumors has been performed, resulting in a new classification that distinguishes nine subtypes of ependymomas and provides more clinical and prognostic information (15).

Overall survival rates in pediatric patients with ependymomas have been reported to range from 40% to 75% (16–19). In South America, the 5-year overall survival rate for patients with intracranial ependymomas is lower than in many high-income countries, frequently not exceeding 45% (7, 20). Gross total macroscopic resection has consistently been reported as the most significant factor associated with increased survival (8, 14, 17, 21). Other factors such as age, location, histological subtype o treatment have been associated with the prognosis in different studies, but with inconsistent results (8, 14, 21–24). Due to the high variability in reported survival rates, identifying the main prognostic factors for these patients treated in low- and middle-income countries (LMIC) should be a priority.

To date, the available information regarding the characteristics and impact of ependymomas in the pediatric population is still limited in South America and Peru. To describe the clinical and demographic characteristics and survival in pediatric patients diagnosed with ependymoma, a review of medical records was conducted for patients treated at the National Institute of Neoplastic Diseases (INEN) between 2012 and 2022.

## Materials and methods

### Settings

Peru has a 31 million people, INEN is a national referral center for cancer, belongs to the Ministry of Health, and serves up to 65% of the national pediatric cancer patients. After completing the approvals by the ethics committee, we conducted a retrospective study based on collecting information from clinical records of patients aged less than 19 years with a diagnosis of ependymoma from 2012 to 2022 at INEN in Lima.

### Statistical analysis

Treatment status was categorized as abandonment if the treatment was suspended for 30 or more days due to non-medical reasons. Time intervals from symptoms to diagnosis and from diagnosis to outcome were evaluated. The date of diagnosis was considered as evidence of a brain tumor on computed tomography (CT) or magnetic resonance imaging (MRI). Alternatively, the date of the first surgical intervention was used if this was unavailable.

Qualitative variables were described using frequencies and percentages, while quantitative variables were described using measures of central tendency and dispersion. The association of categorized data was determined using the chi-square test, and the magnitude and direction were expressed using relative risks. Survival analysis was conducted using Kaplan-Meier curves, and comparisons were made using the Log-Rank test. Multivariate analysis of prognostic factors was performed using the Cox proportional hazards test. A bilateral p-value of <0.05 was considered significant. Statistical analysis was performed using STATA 17^®^ software.

## Results

### Epidemiological profile

**Figure 1:**
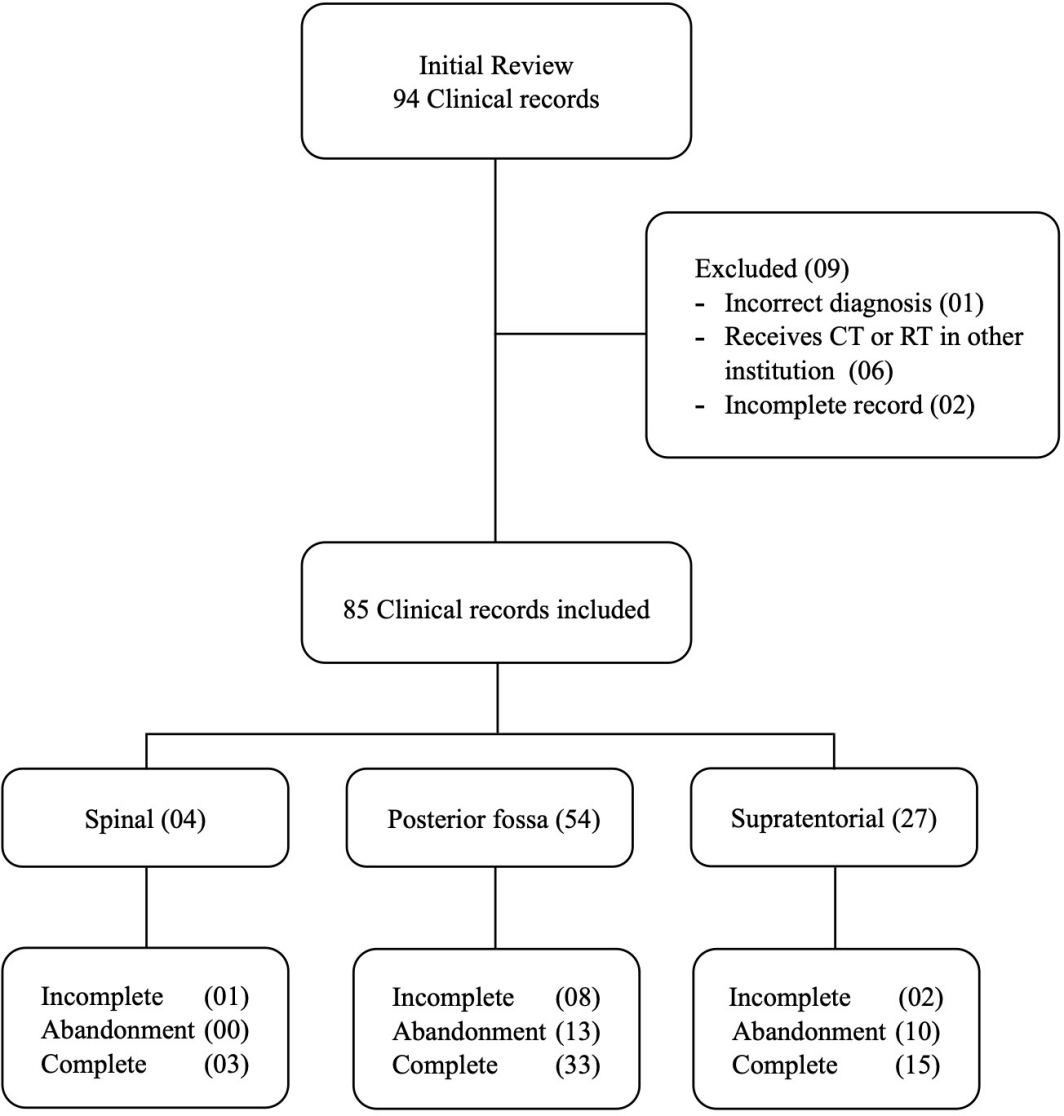
Flow diagram of included medical records.

Ninety-four clinical records were assessed. Nine patients were excluded due to receiving radiotherapy or chemotherapy at another institution (n= 06), incomplete medical records (n= 02) or an incorrect diagnosis (n= 01). Eighty-five medical records were included for the analysis. (**Figure 1**) The baseline characteristics of the patients and tumors are described in **Table 1**. The mean age of the patients was 6.94 years (range 1-19 years), and the prevalence was higher in male patients (male-to-female ratio of 1.5:1). The most common location was the posterior fossa (n=54; 63.53%), and the most frequent histological subtype was anaplastic ependymoma (n=45; 52.94%).

**Table 1:** Patient baseline characteristics.

	Median	Range
Age at diagnosis (years)	6.94	1-19
	Frequency (n)	Percent (%)
Age		
0-2 years	20	23.52
3-10 years	43	50.58
11-19 years	22	25.88
Gender		
Female	34	40
Male	51	60
Location		
Supratentorial	27	31.76
Posterior Fossa	54	62.35
Spinal	4	4.71
Histology (Grade)		
Myxopapillary (2)	2	2.35
Classic Ependymoma (2)	38	44.71
Anaplastic (3)	45	52.94
Presence of Metastasis		
Yes	10	11.76
No	75	88.24
Extent of resection		
Biopsy	1	1.18
Subtotal	55	64.71
Total	27	31.76
Not specified	2	2.35
Treatment received		
Neurosurgery	85	100
Radiotherapy	65	76.47
Chemotherapy	26	30.29

Complete disease staging, consisting in a craniospinal magnetic resonance imaging (MRI) and lumbar puncture, was performed for 32 patients (37.64%). For 35 patients (41.17%) only a craniospinal MRI was performed and, for 3 patients, (3.53%) only a lumbar puncture was performed. Staging studies were not documented for 15 patients (17.65%). Ten patients (11,76%) had metastasis, all located in the spine. Patients in whom a lumbar puncture date was recorded (n=35; 41.17%), the median time between the surgical resection and the lumbar puncture was 62 days (IQR 41.5-144; range 27-733). Patients in whom a postoperative MRI was recorded (n=67; 78-8%), the median time between surgical resection and MRI was 64 days (IQR 36.-103.3; range 8-420).

### Clinical characteristics

The clinical characteristics are described in **Table 2**. The median duration of symptoms until diagnosis was 3 months (IQR 2-5; range 0-40). The most common symptoms were headache (n=49; 57.65%), nausea and vomiting (n=40; 47.06%), and ataxia (n=24; 28.24%). Patients with supratentorial ependymoma were more likely to present with hemiparesis (RR=12.88, 95% CI: 1.63-101.85, p=0.0014); those with posterior fossa location had a higher likelihood of ataxia (RR=2.87, 95% CI: 1.07-7.63, p=0.0448), and those with spinal location had a higher likelihood of neck pain (RR=20.25, 95% CI: 3.76-109.01, p<0.0001) and paraparesis (RR=40.5, 95% CI: 4.57-358.43, p<0.0001). Additionally, it was observed that seizures occurred exclusively in the supratentorial location, while dizziness was only reported in the posterior fossa location. There was also an association between age and symptom presence. Headache was more frequently reported in patients aged 3 years or older compared to those under 3 years of age (RR 2.20, 95% CI: 1.10-4.40, p=0.0042). On the contrary, psychomotor development abnormalities were only described in patients under 2 years of age.

**Table 2:** Clinical manifestations according to tumor location.

Symptoms	Location		
	Supratentorial (n=27)	Posterior Fossa (n=54)	Spinal (n=4)
Headache	15 (55.6)	34 (64.2)	0 (0)
Nausea and vomiting	10 (37)	29 (54.7)	0 (0)
Ataxia	3 (11.1)	19 (35.8)	1 (25)
Hemiparesis	6 (22.2)	0 (0)	1 (25)
Visual problems	3 (11.1)	3 (5.66)	0 (0)
Muscle weakness	0 (0)	4 (7.5)	1 (25)
Neck pain	0 (0)	2 (3.8)	2 (50)
Somnolence	2 (7.4)	1 (1.9)	0 (0)
Paraparesis	0 (0)	1 (1.9)	2 (50)
Psychomotor development alterations	1 (3.7)	2 (3.8)	0 (0)
Dysarthria	1 (3.7)	2 (3.8)	0 (0)
Seizures	2 (7.4)	0 (0)	0 (0)
Dizziness	0 (0)	2 (3.8)	0 (0)
Macrocephaly	1 (3.7)	1 (1.9)	0 (0)
Others	0 (0)	7 (13.2)	3 (75)
Not specified	3 (11.1)	6 (11.3)	0 (0)

### Treatment

In Peru, the majority of surgeries are done in General Pediatric Institutes for patients less than 19 years. Complete safe resection and adjuvant focal radiotherapy is the standard of care. Patients less than 3 years old were treated with different approach of chemotherapy until a second look surgery is possible or until they reach 3 years old at which point radiotherapy is administered. At INEN, the decision of administering radiotherapy and the specific radiation dose is contingent upon the tumor’s location, histological grade, and the extent of resection.

All patients underwent a neurosurgical procedure (n=85; 100%). The most first surgical interventions in pediatric patients with ependymomas took place in General Pediatric Institutes (n= 57, 67%), followed by General Hospitals (n= 17, 20%) and the remaining at INEN (n= 11, 13%). In the first procedure, gross total resection of the tumor was achieved in 27 patients (31.76%); subtotal resection in 55 patients (64.71%), and only a biopsy was performed in 1 patient (1.18%). The extent of surgery could not be determined in 2 cases due to limited information in the medical records. Among patients with subtotal resection, 7 underwent second-look surgery. In the second procedure, one patient achieved gross total resection, and in a third procedure, two patients did. There was no association between the location and extent of resection (chi-square 4.73, p=0.578).

Adjuvant therapy is described in **Table 3**. A total of 65 patients received radiation therapy (76.47%). Patients with supratentorial ependymomas received an average dose of 56.81 Gy (SD 2.91; range 53.60-60.00). Those with posterior fossa location received 55.76 Gy (SD 2.73; range 50.00-60.00), and those with spinal location received 46.10 Gys (SD 5.33; range 39.00-50.40). Four patients did not complete radiation therapy due to abandonment (n=3, 4.61%) or death (n=1, 1.53%). One patient with supratentorial ependymoma received a limited dose of 40 Gys due to the presence of multiple lesions. The median time between the first surgical intervention and the first radiotherapy session was 151 days (IQR 67-191, range 25-868). The median interval between the first surgical resection and radiotherapy initiation in younger than 3 years was 194.8 days (IQR 95-268, range 47-407), while in patients older than 3 years was 143.65 days (IQR 41-448, range 25-868).

**Table 3:** Adjuvant therapy.

	Adjuvant therapy			
	RT n (%)	CT n (%)	RT + CT n (%)	None n (%)
Age (years)				
1-2	5 (11.1)	5 (100)	5 (25)	5 (33.3)
3 or more	40 (88.9)	0 (0)	15 (75)	10 (66.6)
Histology (Grade)				
Myxopapillary (2)	0 (0)	0 (0)	1 (5)	1 (6.6)
Classic Ependymoma (2)	22 (48.9)	2 (40)	7 (35)	7 (46.7)
Anaplastic (3)	23 (51.1)	3 (60)	12 (60)	7 (46.7)
Location				
Spinal	3 (6.6)	0 (0)	1 (5)	0 (0)
Posterior fossa	27 (60)	2 (40)	15 (75)	10 (66.6)
Supratentorial	15 (33.3)	3 (60)	4 (20)	5 (33.3)

CT, Chemotherapy; RT, Radiotherapy; RT + CT, Radiotherapy and Chemotherapy.

Chemotherapy was administered to 26 patients (30.59%) and the most common regimen consisted of 8 cycles of vincristine and cyclophosphamide alternating with etoposide and carboplatin (n=19, 73.07%). In patients under 3 years of age, chemotherapy was administered as a bridge therapy for a second surgical intervention (n=3, 30%) or radiotherapy (n=7, 70%). Of the latter group, 5 patients abandoned treatment before starting radiotherapy. In patients over 3 years of age, chemotherapy was administered following radiotherapy (n=15, 93.75%) or as a bridge therapy for a second surgical intervention (n=1, 6.25%).

A significant association was found between age and the treatment regimen received (chi-square 20.93, p<0.001). Chemotherapy as a sole adjuvant was used exclusively in patients under 3 years of age (n=5, 100%), while adjuvant radiotherapy was used mostly in patients over 3 years (n=30, 88,9%). There was no association between the treatment regimen and histological classification (chi-square 3.97, p=0.86) or location (chi-square 10.50, p=0.31).

Overall, 51 patients (60%) completed treatment, 23 patients (27.06%) abandoned the treatment, and 11 patients (12.94%) did not complete it due to clinical deterioration or death. An association was found between treatment adherence and patient age. Patients aged 3 years or younger were more likely to abandon treatment (RR=2.5, 95% CI: 1.30-4.81, p=0.0083). Additionally, patients under 3 years of age were less likely to complete the treatment (RR 0.64, 95% CI: 0.34-1.06, p=0.0368). There was no association between treatment adherence and location (p=0.515), histological subtype (p=0.432), or province of origin (p=0.31).

### Outcome

During the follow-up period, local recurrence was observed in 18 patients (21.18%). One patient with a primary supratentorial location experienced recurrence in the spinal cord (1.18%). The average time between the first neurosurgical resection and recurrence was 21.07 months (IQR: 10.43-28.13, range 6.87-54.5 months). No association was found between the treatment received and recurrence (chi-square: 8.41, p=0.209). Sequelae were present in 30 patients. The most frequent sequelae were visual problems (n=10, 33.3%), hemiparesis (n=7, 23.3%), facial paralysis (n=4, 13.3%), gait difficulties (n=5, 16.7%), endocrinological problems (n=2, 6.7%), monoparesis (n=2, 6.7%), nasogastric tube usage (n=3, 10%), and tracheostomy tube (n=2, 6.7%). At the end of the follow-up period, 42 deaths were documented (49.4%).

### Survival analysis and prognostic factors

**Figure 2:**
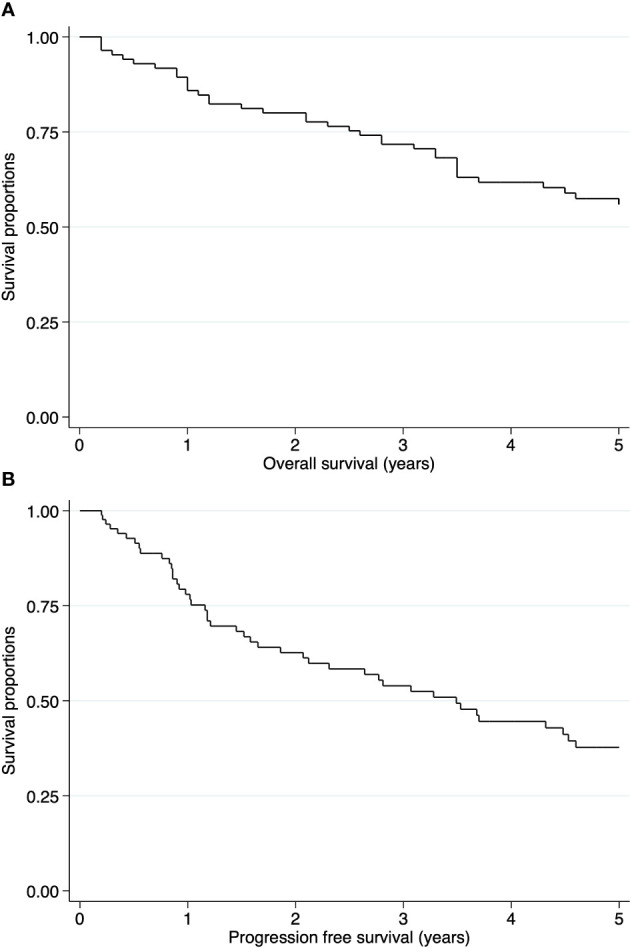
Kaplan-Meier curves of pediatric ependymomas treated at INEN. **(A)** Overall survival (OS) of all cases. **(B)** Progression free survival (PFS) of all cases.

**Figure 3:**
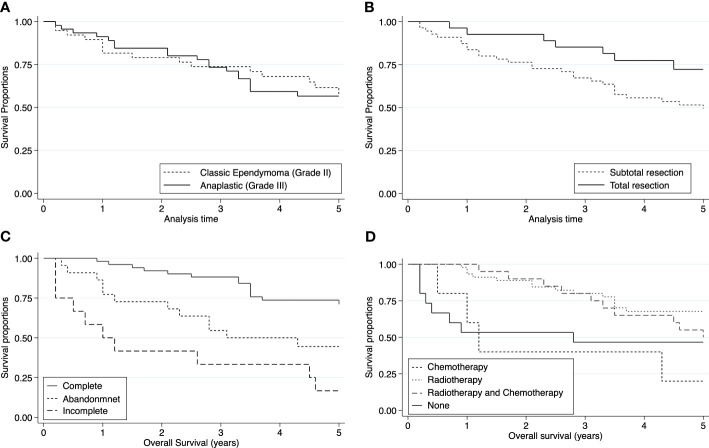
Kaplan Meier curves of pediatric ependymomas treated at INEN. **(A)** Histology subtype was not associated with OS (p=0.99). **(B)** Extension of resection was significantly associated with 5-year OS (p=0.03). **(C)** Treatment adherence was significantly associated with 5-year OS (p<0.001). **(D)** Adjuvant treatment was significantly associated with 5-year OS (p=0.039).

The median follow-up time was 51.6 months. The 5-year OS and PFS rates were 55.89% (95% CI: 44.28-65.99) and 37.71% (95% CI: 26.21-49.16), respectively (**Figure 2**). In the intracranial ependymoma group, the 5-year OS rate was 56.35%, while in the spinal ependymoma group, it was 50%. In the univariate analysis, histologic subtype (p=0.002), the extension of resection (p=0.019), treatment adherence (p=0.0001) and adjuvant treatment (p=0.03) were significantly associated with the OS (**Figure 3**, **Table 4**).

**Table 4:** Univariate analysis of prognostic factors of Progression Free Survival (PFS) and Overall Survival (OS) at 5-year follow up.

Prognostic factors		Frequency (%)	5-year		5-year	
			PFS ± SD (%)	P value	OS ± SD (%)	P value
Age (years)	0-2	21	30.3 ± 11.0	0.477	52.0 ± 11.9	0.94
	3-19	64	40.3 ± 7.0		57.0 ± 6.3	
Extent of resection	Total	27	56.0 ± 11.3	0.032	72.3 ± 9.1	0.03
	Subtotal	55	29.7 ± 6.9		49.3 ± 6.9	
Histology (Grade)	Anaplastic (3)	45	33.3 ± 7.8	0.49	56.6 ± 7.6	0.99
	Classic (2)	38	46.7 ± 9.4		57.7 ± 8.5	
Adjuvant therapy	Radiotherapy (RT)	45	49.6 ± 8.5	0.019	67.7 ± 7.2	0.039
	Chemotherapy (CT)	5	20.0 ± 17.9		17.9 ± 0.8	
	RT + CT	20	31.6 ± 11.0		50.0 ± 11.2	
	None	15	26.0 ± 14.2		46.7 ± 12.9	
Adherence	Complete	51	55.4 ± 8.0	<0.001	71.1 ± 55.9	<0.001
	Abandonment	23	15.2 ± 8.1		42.5 ± 10.5	
	Incomplete	11	10.3 ± 9.8		18.2 ± 11.6	
Presence of Metastasis	Yes	10	11.3 ± 10.6	0.046	30.0 ± 14.5	0.083
	No	75	41.5 ± 6.5		59.2 ± 5.9	
Recurrence	Yes	19	N.A.	<0.001	37.5 ± 12.1	0.078
	No	66	52.1 ± 7.1		60.1 ± 6.2	

In the multivariate analysis, age less than 3 years (HR=0.17, 95% CI: 0.04-0.64, p=0.009) and completion of treatment (HR=0.25, 95% CI: 0.09-0.72, p=0.010) were significantly associated with higher OS. On the contrary, the presence of metastasis (HR=3.66, 95% CI: 1.47-14.46, p=0.008), adjuvant treatment with chemotherapy alone (HR=4.79, 95%CI: 1.18-34.89, p=0.031), and disease recurrence (HR=4.90, 95% CI: 1.78-13.45, p=0.002) were associated with lower OS (**Table 5**).

**Table 5:** Multivariate analysis of 5-year OS prognostic factors.

Characteristic	Hazard ratio (IC 95%)	*p*
Age less than 3 years	0.17 (0.04-0.64)	0.009
Adjuvant chemotherapy only	6.41 (1.18-34.89)	0.031
Complete treatment	0.25 (0.09-0.72)	0.010
Presence of metastasis	3.66 (1.47-14.46)	0.008
Recurrence	4.90 (1.78-13.45)	0.002

## Discussion

Our sample is highly representative of the actual incidence of pediatric ependymomas described by Lima’s Cancer Registry 2013-2015 (2). The outcomes of our cohort are similar to what has been reported in the region. A study conducted in Mexico, which included patients under 17 years old diagnosed with ependymoma, describes a 5-year OS of 58.04% (19). In South America, the 5-year OS for patients with intracranial ependymomas has not exceeded 45% (5, 20). In Peru, a study conducted on patients with spinal ependymomas found a 5-year OS of 85.7% (21) in the pediatric subgroup, while a study on pediatric patients with intracranial ependymomas reported a 5-year OS of 70% (22). On the contrary, studies conducted in the United States and Japan report a 5-year OS close to 75% (12, 18).

A lower survival rate in cases of spinal ependymomas compared to other reports (25) is likely due to a small sample size, with only four patients included in our series. A lower survival rate in developing countries compared to developed countries could be attributable to greater difficulty in accessing the healthcare system, longer waiting times, and lower infrastructure and equipment (26).

The diagnosis of pediatric ependymomas pose a significant challenge for healthcare providers as clinical manifestations of brain tumors are nonspecific and often occur in other, more frequent, pathologies (27–32). Additionally, age plays an important role in the identification of these symptoms. For example, in our cohort, headache was less frequently reported in patients under 3 years of age, probably due to the patient’s inability to accurately express their discomfort and caregivers’ interpretation of the symptom. Psychomotor development disorders were likely limited to patients under 2 years of age, as ataxia or dysarthria may have been interpreted as an inability to walk or speak by primary care physicians.

The classification of ependymomas has undergone multiple changes in the last decade, with a current focus on molecular characteristics. The clinical-pathological utility of histological classification has been contradictory and lacks reproducibility due to high inter-observer variability (14, 33–35). The molecular component of the current classification can potentially provide prognostic information and contribute to therapeutic decision-making, which is still under investigation. This is reflected in current guidelines, which recommend including molecular characteristics in the classification of ependymomas (36, 37). However, performing this classification requires expensive and less available laboratory techniques, limiting its application in low- and middle-income countries (9).

The standard treatment is considered to be maximal safe neurosurgical resection followed by radiation therapy as they have been associated with improved disease-free and progression-free survival (8, 10, 36, 38, 39). Total macroscopic resection has been identified as the most important independent prognostic factor (21), even considered sufficient in some centers for grade 2 supratentorial ependymomas (40–42). In addition to surgery, postoperative radiation therapy at doses of 54-59.4 Gys is considered the standard treatment for non-metastatic ependymomas to reduce the rate of local recurrence (8). Nonetheless, the benefits of these treatments did not reach statistical significance in our cohort. These results may be due to unmeasured factors such as tumor size at the time of initial intervention and delays in starting radiation therapy.

A study conducted in Peru that included patients of ages 3 to 15 years with the diagnosis of medulloblastoma identified that a delay greater than 30 days in the initiation of radiotherapy after surgery was associated with a poor prognosis (43). In our cohort, only 1 patient commenced radiotherapy in the first 30 days after surgery, which may have limited the statistical significance of this factor. Factors such as insufficient healthcare infrastructure and equipment, lack of appointment availability or socioeconomic factors to attend the appointments for the disease staging could potentially contribute to delays in the initiation of radiotherapy. However, being a retrospective study, the precise factors contributing to the delays in the study timeline cannot be determined with certainty.

In spite of their well described benefits, it’s important to acknowledge that complete resection can only be achieved in 50-80% of cases due to inaccessible locations and the risk of neurovascular injury (44). In patients in whom total tumor resection was not achieved, the main limiting factor for reoperation is the risk of increased morbidity. In Peru, the lack of specialized multidisciplinary teams (45), such as pediatric neurosurgeons (46) and pediatric ICU doctors (47), in addition to equipment constraints, or the lack of specialized postoperative care such as nutritional support and rehabilitation specialists, could account for the low percentage of total resections performed.

Radiation therapy can cause adverse effects, affecting cognitive development and, in some cases, the growth of patients, which is more pronounced in children under 3 years of age (38). Historically, efforts have been made to limit radiation therapy in children under 3 years by administering chemotherapy to delay the start of radiation therapy or even replace it (8, 11, 38, 48–50). At our institution, patients under 3 years of age were less likely to receive radiation therapy compared to those over 3 years old. This finding was also described in a study conducted by the University of California, San Francisco, which showed that only 30% of patients under 3 years old with intracranial ependymomas received radiation therapy, compared to 82% of patients over 3 years old (18). However, multiple studies that have shown that delaying radiation therapy in children under 3 years results in a worse prognosis (49, 51), as well as replacing it with postoperative chemotherapy (16, 52). Furthermore, radiation therapy has already been safely used in patients as young as one year old (49, 50, 53–55), so there should be no restriction on this treatment in this group of patients.

The evidence regarding chemotherapy usefulness in pediatric ependymomas is still controversial as it has not consistently translated into improved overall survival and is associated with grade III or IV toxicity in various organ systems in many cases (11), limiting its application and long-term adherence.

Various studies support the adjuvant use of chemotherapy in different scenarios, including chemotherapy combined with radiation therapy in patients with subtotal and near-total resection (53), chemotherapy to delay or replace radiation therapy in children under 3 years (11), or as a bridging therapy for a second intervention (52, 53, 56). On the other hand, multiple studies have failed to demonstrate an advantage in administering chemotherapy in different regimens (22, 57–59). In our study, adjuvant chemotherapy alone was associated with significantly lower survival, highlighting the importance of radiotherapy in the treatment of pediatric ependymomas.

Treatment adherence in pediatric oncology patients poses a significant challenge and plays a crucial role in achieving desired outcomes. Despite the heterogeneity in the treatments received, adherence emerged as a significant prognostic factor in our study, with higher survival rates observed among patients who completed the treatment. Factors influencing treatment adherence include but are not limited to, socioeconomical, patient-related and healthcare-related factors. The presence of other siblings, transportations issues or financial constraints are among the factors likely to limit the adherence of cancer patients in LMIC (60). A study conducted in two tertiary referral centers for the treatment of pediatric patients in Peru identified that socioeconomic factors such as living in a rural household or having an informal employment significantly impacted the abandonment rate in pediatric solid tumors (61). Further studies focusing on identifying factors contributing to suboptimal adherence in pediatric patients with central nervous system tumors are needed in order to address this issue with public health strategies.

Delays in the diagnosis of pediatric brain tumors can lead to disease progression; as reported in pediatric low grade gliomas (62), and decreased survival (63). Brain tumors factors, such as the histology and location, influenced the duration of the prediagnostic symptom interval (63–65). Caregiver factors such as the education level of the parents, previous knowledge of the disease and cultural beliefs were identified as factors that impacted the time to diagnosis (66, 67). In LMIC, healthcare factors can significantly contribute to delays in the diagnosis and initiation of treatment of pediatric patients with brain tumors. The distance to the health center, it’s complexity and the availability of specialists have determined the time to diagnosis in different studies (66, 67).

Identifying factors related to patients who were unable to complete treatment due to deterioration in their clinical condition would help in risk stratification and prioritizing the treatment of this group of patients. Unmeasured factors such as the preoperative status of the patient or tumor size at the time of diagnosis may be related to this outcome.

Contrary to various reports, being under 3 years of age was identified as a protective factor in our study population. These findings are most likely to be related to a low sample of patients receiving the standard treatment associated with a high abandonment rate. Studies evaluating a larger sample of patients younger than 3 years should be performed in order to adequately assess prognostic factors in this age group.

Our study was conducted at a single center convering 65% of the pediatric cancer population diagnosed in Peru. However, some limitations were identified. There is potential for selection bias, given that the majority of patients were insured under the Sistema Integral de Salud (SIS), which primarily serves the underserved population. To obtain a more accurate picture of the reality in our country, it would be necessary to include institutions that serve patients with other types of insurance, corresponding to the remaining 35% of the population. Secondly, being a retrospective cohort based on medical records, the signs and symptoms documented relied entirely on their accurate registration. Problems related to patient follow-up could be avoided as the medical records in our institution are integrated with the National Death Information System (SINADEF). This integration has allowed us to obtain precise information about dates of death and the current status of patients.

## Conclusions

The clinical and demographic characteristics of our patient series are similar to those reported in the literature. The main favorable prognostic factor identified was the completion of treatment. On the contrary, adjuvant chemotherapy alone, the presence of metastasis, and disease recurrence were identified as poor prognostic factors. Histological classification did not provide prognostic information in this cohort. Studies incorporating molecular classification will be necessary to determine the epidemiology and assess prognostic utility. Special focus should be directed to understand the factors influencing a timely diagnosis, early referral, and optimal treatment in patients with ependymoma treated at INEN. Likewise, similar studies must be conducted to assess the prognostic factors of other brain tumors and childhood cancers in our institution.

## Data availability statement

The raw data supporting the conclusions of this article will be made available by the authors, without undue reservation.

## Ethics statement

The studies involving humans were approved by Comité Institucional de Ética en Investigación (CIEI) of the Instituto Nacional de Enfermedades Neoplásicas (INEN). The studies were conducted in accordance with the local legislation and institutional requirements. Written informed consent for participation was not required from the participants or the participants’ legal guardians/ next of kin in accordance with the national legislation and institutional requirements.

## Author contributions

EP-RQ: Conceptualization, Data curation, Formal analysis, Investigation, Methodology, Visualization, Writing – original draft, Writing – review & editing. RD-C: Conceptualization, Formal analysis, Methodology, Supervision, Validation, Writing – original draft, Writing – review & editing. TN: Validation, Writing – review & editing. SC-Z: Validation, Writing – review & editing. LO-M: Validation, Writing – review & editing.
